# Synthesis and Modifying Effect of Oligoesters with Reactive Groups Based on Epoxy Aliphatic Resin and Oligoester Dicarboxylic Acids

**DOI:** 10.3390/polym17040433

**Published:** 2025-02-07

**Authors:** Xiangli Meng, Qing He, Tetiana Hryhorenko

**Affiliations:** 1 School of Chemistry and Chemical Engineering, Harbin Institute of Technology, Harbin 150001, China; mengxl2009@hit.edu.cn (X.M.); 13223665300@163.com (Q.H.); 2 Chongqing Research Institute of Harbin Institute of Technology, Chongqing 401120, China

**Keywords:** epoxy resin, dicarboxylic acid, oligoester, reactive modifiers, mechanical properties

## Abstract

The purpose of this research is to study the modifying effect of oligoesters with reactive groups based on epoxy aliphatic resin and oligoesters with dicarboxylic acids with different molecular weights (adipic, sebacic, and tetradecanedioic acids). Adducts of oligoesters with terminal epoxy groups and epoxy resin (ER) were prepared. The structures of the intermediates and modifiers were characterized by FTIR, ^1^H NMR, ^13^C NMR, TGA, DMA, and SEM. The single-phase structure of the modified polymers was confirmed using the DMA, TGA, and SEM methods. It was shown that for modified polymers, a pattern of plastic deformation is observed, in contrast to the brittle destruction of the initial polymer. It has been found that elongation at break, impact strength, work of fracture, and shear strength increase throughout the studied concentration range (at 50% modifier content, elongation at break and shear strength increase by ~450% and ~150%, respectively, compared with an unmodified polymer). The results obtained demonstrate that synthesized modifiers with reactive epoxy groups can contribute to the creation of new cold-cured epoxy materials with an improved complex of properties for various industries.

## 1. Introduction

With the continuous progress of science and technology and the rapid development of various industries, people’s demand for new materials is becoming more and more urgent. The application of polymer materials is particularly prominent in the 21st century, and is widely regarded as the most important result of the development of materials science in the 21st century. One of the most commonly used polymers is epoxy polymers (EP), which is recognized as the most widely used strategic thermosetting material nowadays [1,2].

As an important structural material, epoxy polymers have good chemical corrosion resistance [3], excellent mechanical [4] and adhesive properties [5,6], and high temperature stability [7], and as a result, epoxy materials are used in many fields, such as chemical corrosion prevention [8], transportation [9], aerospace [10], shipbuilding, composite materials [11], new energy, 5G communication, electronic and electrical [12], mechanical manufacturing, engineering construction [13], and other industrial fields and high-tech industries [14].

Despite the above advantages of EP, their significant disadvantages are low impact resistance, vibration resistance, and crack resistance [15,16,17], which sharply limits their use under the action of dynamic loads and thermal cycles. Moreover, the higher the density of the nodes of the polymer mesh of the EP, the lower, as a rule, the resistance to the formation and growth of cracks [18,19,20]. The introduction of inert modifiers (non-reactive plasticizers) does not change the length of these segments [21], and the stoichiometric ratio change between the epoxy resin and the curing agent leads to high defects in the cross-linked polymer [22]. Therefore, the adjustment range of inert modifiers is limited, and the modification of epoxy polymers under cold curing conditions is decreased compared with that of hot curing, so modification for cold curing epoxy resin systems is of great significance and necessity. Currently, one of the most promising methods is the chemical modification of the polymer mesh by using new reactive modifiers with active groups, which can extend the length of the chain segment between the cross-linked nodes [12,23,24,25,26,27,28,29]. Chen et al. [24] synthesized a monoglycidyl silyl etherated eugenol (GSE) as a reactive diluent and modifier for BPA-based epoxy resin. The curing kinetics, thermal, and mechanical properties of the cure epoxy/anhydride system with different BPA weight ratios were investigated. The results indicated that GSE can effectively improve the toughness and thermal decomposition temperature of the epoxy system. Zhang et al. [25] synthesized epoxy-terminated polyurethane (EPU)-modified epoxy resins with different EPU contents. The results indicated that the toughness of the epoxy resin was significantly improved after the addition of EPU.

Therefore, the task of synthesizing new reactive modifiers capable of lengthening the chain and forming a single spatially cross-linked network of covalent chemical bonds in a cured epoxy polymer is relevant.

At the first stage of the study, three oligoesters with terminal carboxyl groups based on dicarboxylic acids with different molecular weights (adipic, sebacic, and tetradecanedioic acids) and diethylene glycol were synthesized. In the second stage, adducts of oligoesters and epoxy resin (ER) with terminal epoxy groups were synthesized. The structures of the intermediates and modifiers were characterized by FTIR, ^1^H NMR, ^13^C NMR, TGA, DMA, and SEM. At the third stage of the study, the mechanical properties of epoxy polymers were determined depending on the content of reactive modifiers.

## 2. Experimental

### 2.1. Materials

Adipic acid, sebacic acid, tetradecanedioic acid, diethylene glycol, diethyleneglycol diglycidyl ether, and diethylene triamine (DETA), all analytically pure, were purchased from Aladdin Reagent Co., Ltd. (Shanghai, China) All the reaction reagents were used directly without additional purification.

A commercially available bisphenol-A type epoxy resin (CYD-128, Export grade, Baling Branch of China Petroleum and Chemical Corporation Limited, Yueyang, Hunan Province, China) was used as the thermosetting matrix and it was cured with diethylene triamine.

### 2.2. Synthesis

This synthesis is divided into two steps: the first step is the synthesis of intermediate oligoesters with terminal carboxyl groups, and the second step is the synthesis of epoxy adducts based on the aliphatic epoxy resin and the oligoesters with terminal carboxyl groups. Next, the synthesis steps will be described in detail.

**Figure 1:**
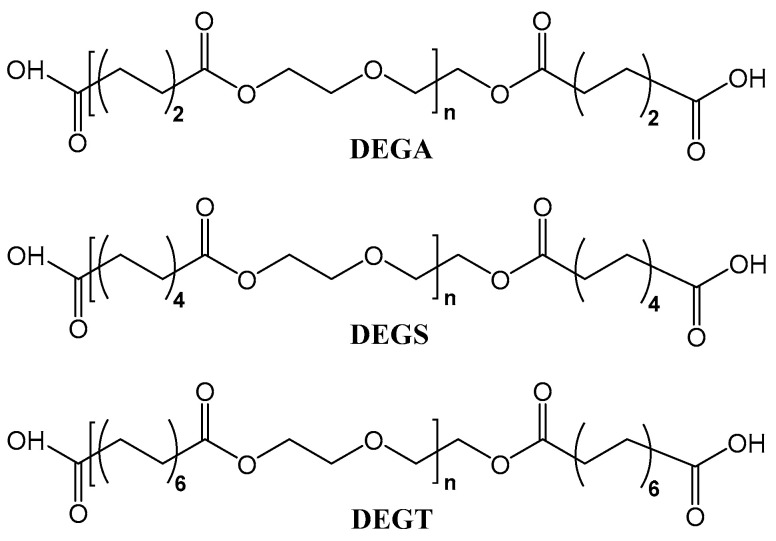
Chemical formulas of synthesized oligoesters with terminal carboxyl groups.

First, the calculated amount of sebacic acid was loaded into a 5-neck flask, equipped with a stirrer, a thermometer, and a reflux condenser with a Dean–Stark trap. The flask was placed in an oil bath. When the temperature in the flask reached 90 °C, the calculated amount of diethylene glycol was loaded. The stirrer and the nitrogen supply were connected when the mixture in the flask became liquid. The polycondensation process was carried out at a temperature of 150–165 °C while controlling the acid number of the reaction mixture using the acid–base titration method. The acid number of the resulting oligoester should be in the range of 130–165 mg KOH/g. The resulting oligodiethylene glycol sebacate (DEGS) with reactive carboxyl groups is placed in a vacuum oven for 2 h and a temperature of 80 °C to remove residual amounts of water. Oligodiethylene glycol adipate (DEGA) and oligodiethylene glycol tetradecanediovate (DEGT) with reactive carboxyl groups were obtained using the above synthesis method. The chemical formulas of the synthesized oligoesters with terminal carboxyl groups are shown in Figure 1.

**Figure 2:**
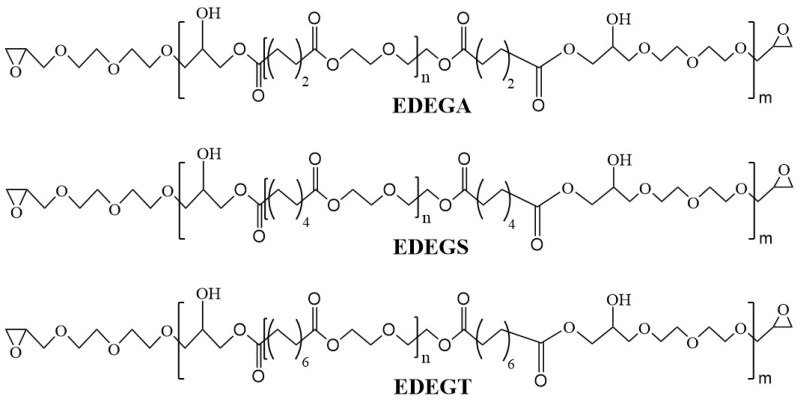
Chemical formulas of synthesized epoxy adducts with terminal oxirane groups.

Second, the calculated amount of oligodiethylene glycol sebacate (DEGS) and the calculated amount of diethylene glycol diglycidyl ether were loaded into a flask, equipped with a stirrer, a thermometer, a reflux condenser with a Dean–Stark trap. The flask was placed in an oil bath. When the temperature in the flask reached 60 °C, the stirrer and the nitrogen supply were connected. The synthesis was carried out at a temperature of 130–155 °C while controlling the acid number of the reaction mixture using the acid–base titration method. The acid number of the resulting adduct should be in the range of 0 mg KOH/g and the epoxy number—in the range of 7–9%. The resulting epoxy adduct (EDEGS) of the aliphatic epoxy resin and DEGS was placed in a vacuum oven for 2 h and a temperature of 80 °C. The epoxy adducts of diethylene glycol diglycidyl ether and the oligoesters based on adipic (EDEGA) or tetradecanedioic (EDEGT) acid were obtained using the above synthesis method. The chemical formulas of synthesized epoxy adducts with terminal oxirane groups are shown in Figure 2.

### 2.3. Modification of the Epoxy Matrix

The synthesized modifiers (EDEGA, EDEGS, and EDEGT) were mixed with epoxy resin (CYD-128) in percentages of 1.5, 5, 10, 20, and 50%. Then, a stoichiometric amount of the hardener diethylene triamine was added with careful stirring. The resulting epoxy compositions were placed in molds treated with an anti-adhesive compound, whose dimensions matched the requirements of the standards for testing. The compositions were cured according to the mode (20 ± 2)°C/150 h, i.e., at room temperature.

### 2.4. Analysis and Testing

#### 2.4.1. Determination of the Acid Number

A total of 0.3–1.0 g of the test sample was placed in the flask, weighted to an accuracy of 0.0001 g (the amount of the sample is determined by the content of the COOH groups), and a mixture of solvents (10 mL of chloroform and 10 mL of neutralized ethyl alcohol) was added and after the dissolution of the sample (15–20 min), it was titrated from a microburet with 0.1 N KOH alcohol solution in the presence of phenolphthalein before the appearance of pink color.

The content of carboxyl groups AN (in %) is calculated by the Formula (1):

(1) $$\mathrm{AN}=\frac{{(V}_{1}-V_{2})\;F\;\times \;0.00561\;\times \;1000}{m},$$

where

V_1_—the volume of 0.1 N KOH alcohol solution consumed for titration of the working sample, mL;

V_2_—the volume of 0.1 N KOH alcohol solution consumed for titration of the control sample, mL;

F—the correction factor of 0.1 N KOH alcohol solution;

0.00561—the titer of 0.1 N KOH solution, g/mL;

m—the mass (g) of the analyzed sample.

#### 2.4.2. Determination of the Epoxy Number

To determine the epoxy number, the sample (<0.1 g) was placed in a 250 mL flask, 5 mL of acetone was added, and it was kept for 8–10 min until the sample dissolved. Then, 10 mL of 0.2 N HCl solution in acetone was added and kept for 30 min, after which it was titrated from a microburet with 0.1 N KOH aqueous solution in the presence of phenolphthalein until a crimson color appeared.

The content of epoxy groups EN (in %) is calculated by the Formula (2):

(2) $$\mathrm{EN}=\frac{{(V}_{1}-{\;V}_{2})\;F\;\times \;0.0043}{m}\times 100,$$

where

V_1_—the volume of 0.1 N KOH aqueous solution consumed for titration of the control sample, mL;

V_2_—the volume of 0.1 N KOH aqueous solution consumed for titration of the working sample, mL;

F—the correction factor of 0.1 N KOH aqueous solution;

0.0043—the number of epoxy groups corresponding to 1 mL of 0.1 N KOH;

m—the mass (g) of the analyzed sample.

#### 2.4.3. Fourier Transform Infrared Spectroscopy (FTIR)

The structure of the oligoesters and epoxy adducts (modifiers) were analyzed using a Nicolet Nexus 670 infrared spectrometer (Thermo Fisher Scientific, Waltham, USA), and the test range was from 500 to 4000 cm^–1^.

#### 2.4.4. Nuclear Magnetic Resonance Spectroscopy (NMR)

The oligoesters and epoxy adducts were analyzed by Bruker-600 MHz nuclear magnetic resonance spectrometer (Bruker Corporation, Billerica, Germany), at room temperature. Dimethyl sulfoxide (DMSO) was selected as the solvent.

#### 2.4.5. Mechanical Properties

According to the GB/T 2567-2021 standard [30], the tensile and bending properties of the modified epoxy polymers were tested by an Instron–1186 universal material testing machine. The elastic modulus (E) was calculated from the slope of the initial portion of the σ–ε curve. The area under the stress–strain curve was considered as a measure of the fracture work.

**Figure 3:**
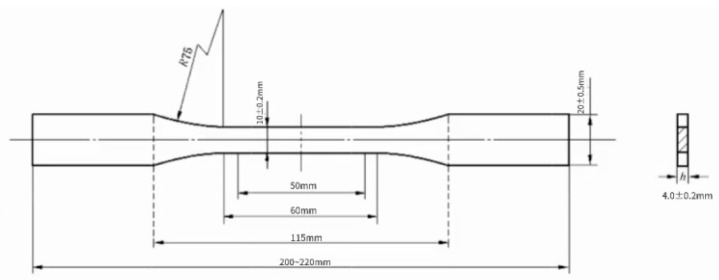
Standard drawing for dimensions of tensile test specimens.

The sample size for tensile testing is shown in Figure 3, and the test is conducted at a loading speed of 2 mm/min.

**Figure 4:**
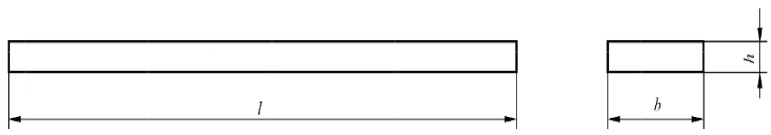
Standard drawing for dimensions of bending test specimens [30].

The sample size for bending testing is shown in Figure 4, and the test was conducted at a loading speed of 2 mm/min. And, the length of the sample l shall not be less than 20 h, the width b shall be 15 mm, and the thickness h shall be 3.0~6.0 mm (with a thickness tolerance of ±0.2 mm for one set of samples). 

**Figure 5:**
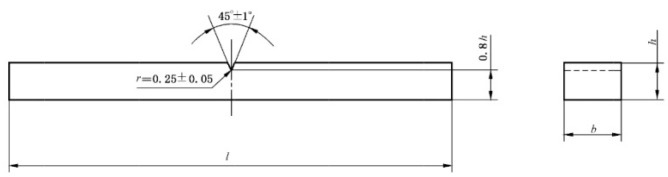
Standard drawing for dimensions of impact test specimens [30].

According to the GB/T 2567-2021 standard [30], a combined testing machine IK10 manufactured by JAY Testing Machine (JAY Instruments, Shanghai, China) was used to measure the impact strength. The impact strength test was conducted using a sample with a V-shaped notch, and the sample size is shown in Figure 5, with a notch depth of 2 mm.

In accordance with the GB/T 7124-2008 standard [31], the adhesive shear strength of aluminum samples was determined using the Instron-1186 universal material testing machine. The surfaces of the gluing samples were treated with sandpaper with a grain size of 80% to a uniform roughness and washed with distilled water. Then, the samples were treated with acetone and dried. The prepared compositions were applied to the surfaces to be bonded and provided uniform pressure during the bonding process. The bonding area was 12.5 × 25 mm. During the test, the loading rate was 2 mm/min.

#### 2.4.6. Dynamic Mechanical Analysis (DMA)

Storage modulus (E′) and tangent of mechanical loss angle (tan δ) were measured on the dynamic thermo-mechanical analyzer of Neutsch, type 242 (FRG). The mode was tensile, and the heating rate was 5 °C/min.

#### 2.4.7. Thermogravimetric Analysis (TGA)

The thermogravimetric analysis was performed with the Q500 thermogravimetric analyzer of TPA in an argon atmosphere with a heating rate of 10 °C/min and a heating range from room temperature to 800 °C.

## 3. Results and Discussion

### 3.1. Characterization of Oligoesters with Terminal Carboxyl Groups

**Figure 6:**
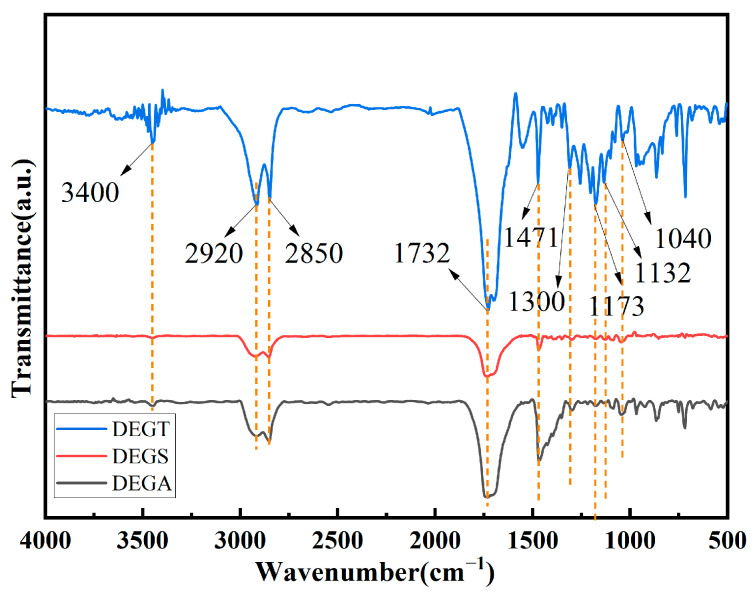
Infrared spectra of synthesized oligoesters.

The structural formulas of the synthesized oligoesters with reactive carboxyl groups are shown in Figure 1. Their chemical structures were confirmed by infrared spectroscopy (Figure 6).

Absorption bands characteristic of aliphatic hydroxyl and carboxyl groups related to O–H stretching vibration were observed in the regions of 3400 cm^–1^. The sharp bands observed at about 2920 and 2850 cm^−1^ can be assigned to CH_2_ antisymmetric and symmetric stretching vibration. The appearance of typical bands for the stretching vibration of C=O bonds inherent to ester and carboxyl groups in the region of 1760–1660 cm^−1^ evidences the esterification reaction occurrence and the presence of terminal carboxyl groups. Two peaks, 1471 cm^−1^ and 1300 cm^−1^, corresponding to the carboxyl C-O-H plane bending vibration and carboxyl C-OH stretching vibration, respectively, indicate the possibility of carboxyl groups in the structure. The peaks located at 1173 cm^−1^ and 1132 cm^−1^ correspond to the asymmetric and symmetric stretching vibrations of the C-O bonds in the ether group, indicating that the ether bond may exist in the structure. The peak of 1040 cm^−1^ is a C-O stretch vibration connected to the alkyl group.

These results showed that these oligoesters with carboxyl groups were successfully synthesized. The presence of carboxyl groups was also proved by acid–base titration. The acid number of the synthesized oligoesters varied in the range of 140–146 mg KOH/g.

**Figure 7:**
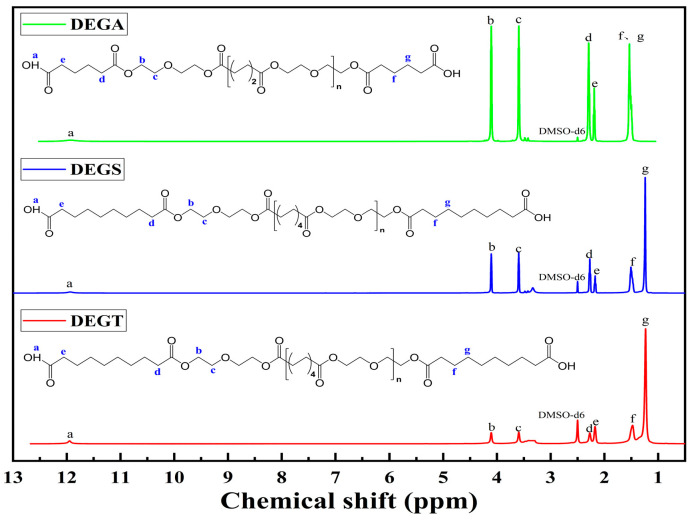
^1^H NMR spectra of synthesized oligoesters.

The chemical structures of the synthesized oligoesters were confirmed by ^1^H NMR (Figure 7). The ^1^H NMR spectra of the synthesized compounds show signals of protons of terminal carboxyl groups in the range of 11.93–11.95 ppm (a) and methylene groups attached to them in the range of 2.17–2.19 ppm (e); methylene groups in α-position to carbon or oxygen atoms of ester groups in the range of 2.27–2.29 ppm (d) and at 4.11 ppm (b), respectively; methylene groups in α-position to ether oxygen atoms at 3.60 ppm (c) and methylene groups in β- and γ-position to carbon atoms of ester groups in the range of 1.47–1.54 ppm (f) and at 1.23 ppm (g), respectively. Because the molecular chain of DEGA is shorter, and the structure is more symmetrical than that of DEGS and DEGT, the atoms are in a similar electronic environment, so the signals of protons (f) and (g) overlap to form a peak. By comparing the information in Figure 1, it is found that it is very consistent with the expected structure of DEGA, DEGS, and DEGT, indicating that the target oligomers have been successfully prepared, and the structure of the synthesized product can be preliminarily judged as shown in Figure 1.

**Figure 8:**
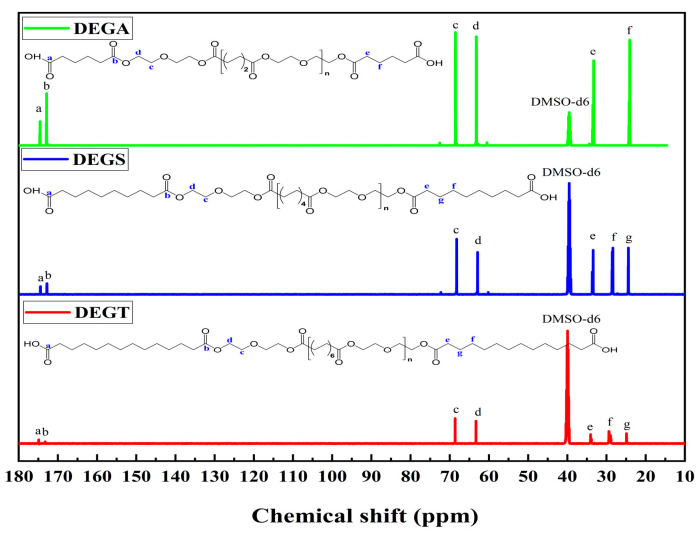
^13^C NMR spectra of synthesized oligoesters.

The chemical structures of the synthesized oligoesters were confirmed by ^13^C NMR (Figure 8). There are signals of ^13^C isotopes in the ^13^C NMR spectra of the compounds related to the terminal carboxyl groups in the range of 174.48–174.55 ppm (a); ester groups in the range of 172.80–173.24 ppm (b); methylene groups attached to ether oxygen atoms in the range of 68.24–68.63 ppm (c); methylene groups in α-position to carbon or oxygen atoms of ester groups in the range of 33.29–33.86 ppm (e) and in the range of 62.91–63.32 ppm (d), respectively; and methylene groups in β- and γ-position to the carbon atoms of ester groups in the range of 28.38–28.55 ppm (f) and in the range of 24.08–24.87 ppm (g), respectively. The peak at 39.52 ppm is the DMSO solvent peak. Because the molecular chain of DEGA is shorter, and the structure is more symmetrical than that of DEGS and DEGT, the atoms are in a similar electronic environment, so the signals of carbon atoms (f) and (g) overlap to form a peak. By comparing the information in Figure 8, it was found that it is very consistent with the expected structure of DEGA, DEGS, and DEGT, indicating that the target polymer has been successfully prepared, and the structure of the synthesized product can be preliminarily judged as shown in Figure 1.

### 3.2. Characterization of Modifiers with Reactive Epoxy Groups

**Figure 9:**
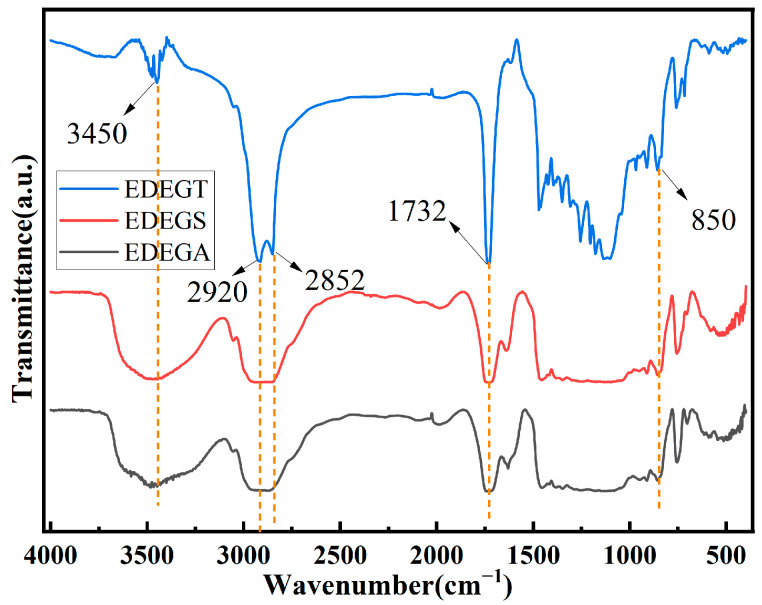
Infrared spectra of modifiers with reactive epoxy groups.

The structural formulas of the synthesized modifiers with reactive epoxy groups are shown in Figure 2. Their chemical structures were confirmed by FTIR (Figure 9).

Characteristic bands belonging to aliphatic hydroxyl O–H stretching vibration were observed in the regions of 3450 cm^–1^. The sharp bands observed at about 2920 and 2852 cm^−1^ can be assigned to CH_2_ antisymmetric and symmetric stretching vibrations. The absorption bands in the region of 1728–1743 cm^–1^ are related to the stretching vibrations of the C=O bonds of ester groups. In addition, the presence of epoxy groups was confirmed by the appearance of typical bands at 850 cm^−1^. These results showed that these modifiers with reactive epoxy groups were synthesized. The presence of epoxy groups was also proved by acid–base titration. The epoxy number of the synthesized oligoesters varied in the range of 7–9.

**Figure 10:**
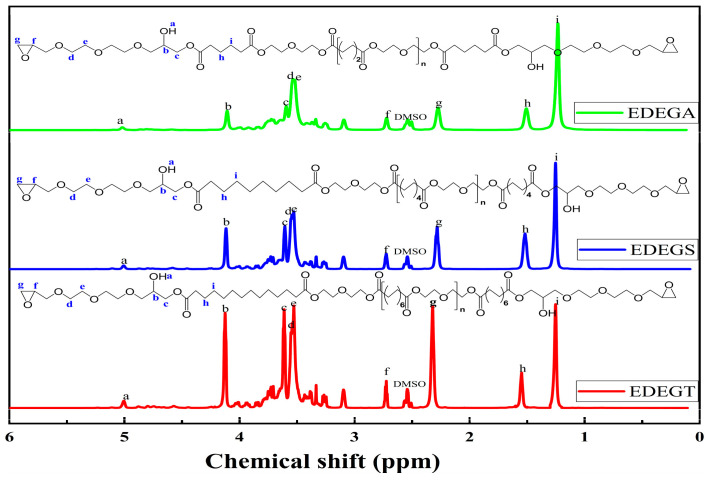
^1^H NMR spectra of modifiers with reactive epoxy groups.

The chemical structures of the synthesized modifiers were confirmed by ^1^H NMR spectra (Figure 10). The ^1^H NMR spectra of the synthesized modifiers show signals of protons of aliphatic hydroxyl groups in the range of 5.01–5.02 ppm (a); methylene groups in α-position to oxygen atoms of hydroxyl and ester groups in the range of 4.11–4.12 ppm (b); methylene groups in α-position to carbonyl groups in the range of 3.59–3.61 ppm (c); ethylene groups of glycidyl(di(oxyethylene))oxide fragments in the range of 3.52–3.54 ppm (d, e); methylene groups in β- and γ-position to carbon atoms of ester groups in the range of 1.51–1.55 ppm (h) and in the range of 1.23–1.28 ppm (i), respectively; and methylene groups of epoxy cycles in the range of 2.72–2.73 ppm (f) and in the range of 2.27–2.32 ppm (g).

By comparing the information in the figure, it is found that it is very consistent with the expected structure of EDEGA, EDEGS, and EDEGT, indicating that the modifiers have been successfully prepared, and the structure of the synthesized modifiers can be preliminarily judged as shown in Figure 10.

**Figure 11:**
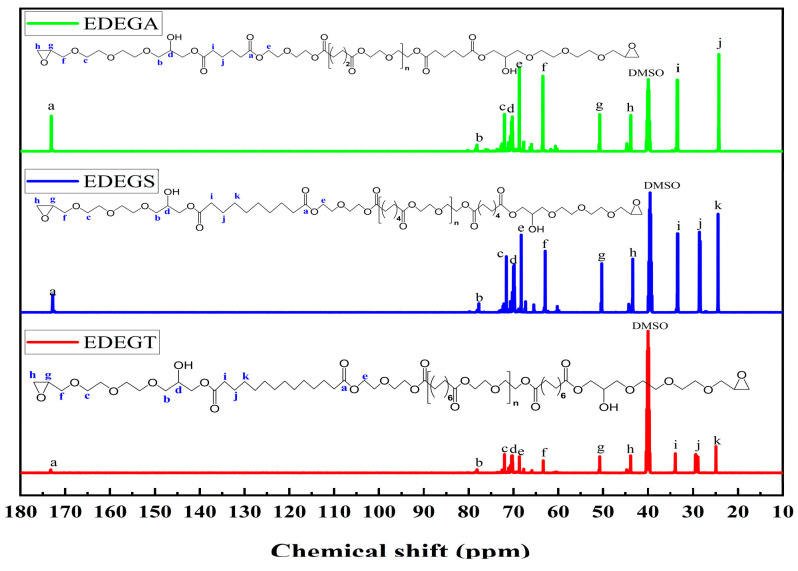
^13^C NMR spectra of modifiers with reactive epoxy groups.

The chemical structures of the synthesized modifiers were confirmed by ^13^C NMR spectra (Figure 11). The ^13^C NMR spectra of the synthesized modifiers contain signals related to the carbonyl groups in the range of 172.80–173.71 (a); methylene groups in α- and β- (attached to ether oxygen) position to aliphatic hydroxyl groups in the range of 70.00–70.41 ppm (d) and in the range of 77.16–78.14 ppm (b), respectively; methylene groups attached to glycidoxide groups in the range of 71.59–72.01 ppm (c); methylene groups in α-position to ester oxygen atoms of carbonyloxyethylene fragments in the range of 68.27–68.69 ppm(e); methylene groups attached to epoxy cycles in the range of 62.93–63.44 ppm (f); tertiary and secondary carbon atoms of epoxy groups in the range of 50.31–50.76 (g) and in the range of 43.39–43.86 ppm (h), respectively; and methylene groups in α-, β-, and γ-position to carbonyl groups in the range of 33.42–33.89 ppm (i), 28.61–29.49 ppm (j) and 24.12–24.45 ppm (k), respectively. By comparing the information in Figure 1, it is found that it is very consistent with the expected structure of EDEGA, EDEGS, and EDEGT, indicating that the target modifiers have been successfully prepared, and the structure of the synthesized product can be preliminarily judged as shown in Figure 2.

### 3.3. Dynamic Mechanical Analysis

**Figure 12:**
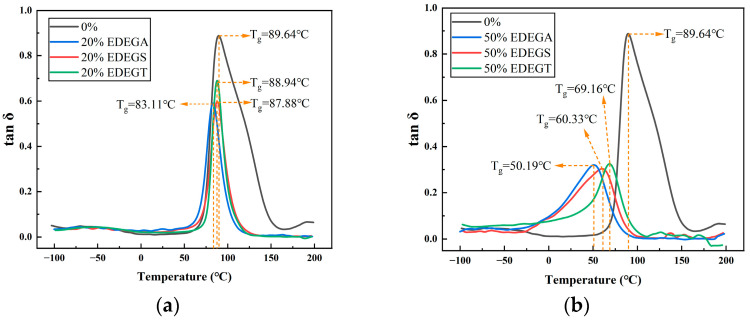
Temperature dependences of the tangent of mechanical loss angle containing 20% (**a**) and 50% (**b**) of the modifying additives.

Figure 12 and Table 1 show the dependence of the tangent of mechanical loss angle (tan δ) on the content of modifiers. At 20%–modifier content in the epoxy polymer, a slight decrease in the glass transition temperature (T_g_) is observed. The intensity of the peak of tan δ and the glass transition temperature decrease depending on the molecular weight of dicarboxylic acid used in the synthesis of modifiers. With an increase in the modifier content to 50%, the glass transition temperature of the modified polymers decreases significantly, as does the intensity of the tan δ peaks, which indicates a significant increase in the free volume in the polymer.

**Figure 13:**
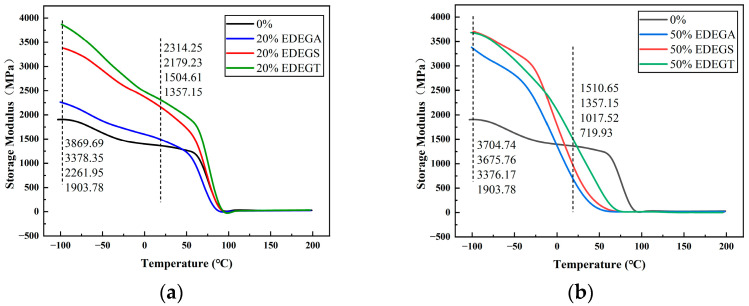
Temperature dependences of the storage modulus containing 20% (**a**) and 50% (**b**) of the modifying additives.

As follows from Figure 13 and Table 2, the addition of modifiers in a wide concentration range contributes to a significant increase in the storage modulus at temperatures below 25 °C. The observed effect is explained by the fact that modifiers have more flexible molecules compared to the base epoxy resin. That is, during curing, the molecules of the modifier are able to form a more densely packed polymer, which in its glassy state (in this temperature range) will have higher mechanical properties, including the storage modulus. The results obtained indicate that the modified polymers are capable of high working capacity at low temperatures [32].

Note also, for all the samples, there is only one peak of tan δ (Figure 12) and one step transition on the curves of storage modulus (Figure 13). This indicates the absence of phase separation [33] and good compatibility between the base epoxy resin and modifiers.

### 3.4. Thermogravimetric Analysis

**Figure 14:**
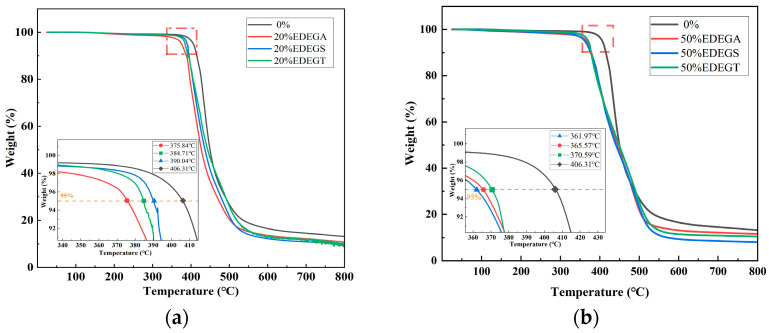
Mass loss curves of epoxy polymers containing 20% (**a**) and 50% (**b**) of modifying additives.

Figure 14 shows the thermal mass loss curves for samples with 20% and 50% modifier content compared to an unmodified polymer. The temperature of 5% mass loss (T_5%_) was accepted as the initial decomposition temperature of the polymer, which decreases with increasing the concentration of the modifier, but this decrease is insignificant. It is shown that T_5%_ decreases by 5.5% with the introduction of 20% modifiers and by about 10% with the introduction of 50% modifiers (Table 3). This makes it possible to use synthesized modifiers in a wide concentration range without fear of a significant decrease in thermal stability. A decrease in the thermal stability of the modified polymers may be associated with a decrease in the cross-linking density [34].

It has been established that polymers containing modifiers have one stage of decomposition, like the original polymer. These results correlate well with the results of dynamic mechanical analysis and indicate the single-phase structure of the modified polymers and the good compatibility of the epoxy resin and modifying additives.

### 3.5. Phase Morphology (SEM)

**Figure 15:**
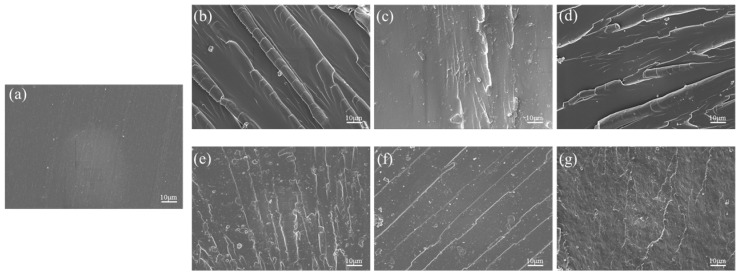
SEM images of fracture surfaces after determining impact resistance modified samples: 0% (**a**), 20% EDEGA (**b**), 20% EDEGS (**c**), 20% EDEGT (**d**), 50% EDEGA (**e**), 50% EDEGS (**f**), and 50% EDEGT (**g**).

The morphology of the surface of the modified epoxy polymers after determining the impact strength was studied using SEM. Figure 15 shows images of sections of the epoxy polymers after impact destruction, containing various amounts of the synthesized modifiers. The results of the study showed no signs of phase separation for all the samples, which is in good agreement with the results of DMA and DTA. The single-phase structure of the modified polymers indicates the possibility of co-curing CYD-128 epoxy resin and a reactive modifier with an amine hardener.

Figure 15a clearly shows that the fracture surface after impact for the unmodified epoxy polymer was without obvious defects, rather smooth, with thin stripes. Such a surface pattern is typical for brittle fractures [34,35]. In contrast, the surfaces of epoxy polymers modified with oligoesters with reactive epoxy groups appear more heterogeneous (rough). Such a pattern (Figure 15b–g) is observed during plastic deformation [36], which gives reason to believe that the fragility of the original epoxy matrix was reduced by introducing flexible chains of modifiers into the mesh of the original epoxy resin.

### 3.6. Mechanical Properties

**Figure 16:**
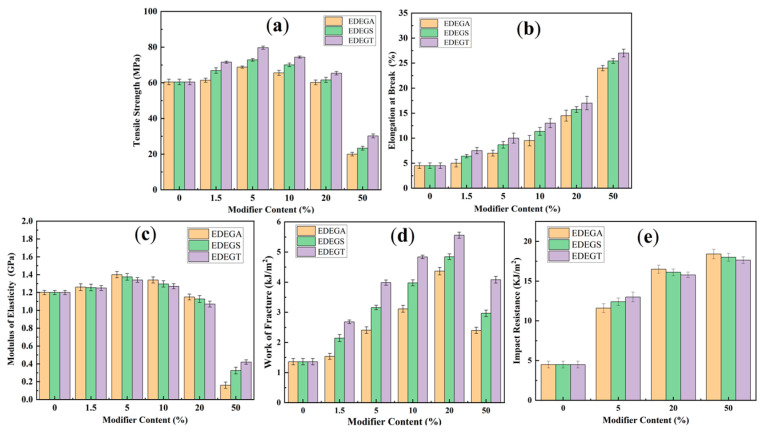
The effect of the concentration of modifiers on the mechanical properties: tensile strength (**a**), elongation at break (**b**), modulus of elasticity (**c**), work of destruction (**d**), and impact resistance (**e**).

Figure 16 shows the dependence of the tensile strength, elongation at break, modulus of elasticity, work of fracture, and impact resistance on the number and chemical structure of the introduced modifiers. It can be seen that the concentration dependences of the tensile strength are extreme (Figure 16a), and the position of the maximum and its intensity depend on the molecular weight (chain length) of dicarboxylic acid, which was used in the synthesis of the modifiers and increases in the series: tetradecanedioic > sebacic > adipic acids. At the same time, the tensile strength at the optimal amount of modifier increases by 8% (EDEGA), 20% (EDEGS), and 31% (EDEGT), respectively, compared to a polymer that did not contain a modifier.

Elongation at break (Figure 16b) increases monotonously throughout the studied range of modifier concentrations in the polymer and increases by 11%(EDEGA), 42% (EDEGS), and 66% (EDEGT) at a modifier concentration of 1.5% to 433% (EDEGA), 422% (EDEGS), and 500% (EDEGT) with 50% modifier content compared to an unmodified polymer.

In Figure 16c, it can be seen that the introduction of the modifiers made it possible to maintain the values of the modulus of elasticity of modified polymers in the concentration range up to 50%, and in the field of small additives of the modifier (1.5–10%) even increase it by 11% (EDEGA), 8% (EDEGS), and 5% (EDEGT). In this case, the magnitude of the effect also depends on the length of the dicarboxylic acid chain, but in this case, the modifier synthesized on the basis of adipic acid (with the shortest chain) shows the greatest increase in the modulus of elasticity.

Due to the simultaneous increase in tensile strength and deformation at break, the work of fracture (A_f_) of the modified polymers significantly increases throughout the studied range of modifier concentrations (Figure 16d). Considering the good correlation between the values of A_f_ and the specific impact strength [37], as well as between the impact shear index [38], which characterizes the performance of the polymer directly in the adhesive joint under the action of dynamic loads, it can be assumed that the use of synthesized modifiers will increase the impact resistance, vibration resistance, and crack resistance of epoxy polymers during their operation both in block samples and in an adhesive joint. The results of direct measurement of impact resistance (Figure 16e) show that an increase in this indicator is observed throughout the studied concentration range. However, as opposed to the work of fracture, the impact resistance is practically independent of the molecular weight of dicarboxylic acids used in the synthesis of modifiers.

**Figure 17:**
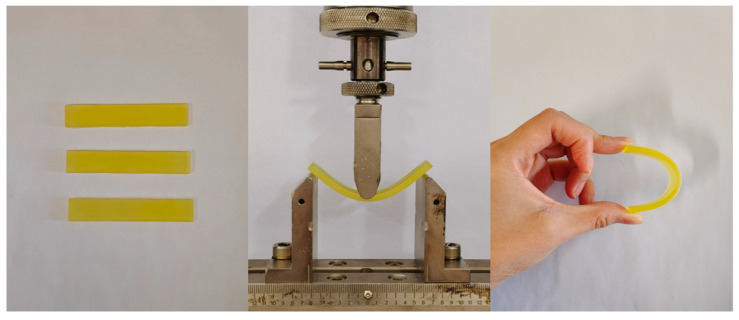
The photos of the samples after bending testing.

The synthesized modifiers increase the bending strength in the concentration range of 1.5–10% (Table 4). Unfortunately, due to the high deformation capacity of the samples, we were unable to obtain the results of the bending strength tests with a modifier content of more than 20% (photo). These samples were not destroyed in this concentration range (Figure 17).

**Figure 18:**
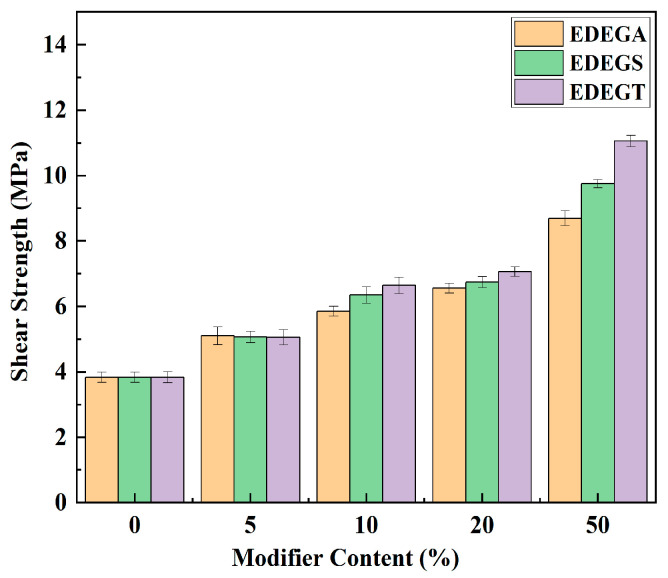
The effect of the concentration of modifiers on the shear strength.

The synthesized modifiers have a positive effect on the adhesive properties. As shown in Figure 18 the shear strength of aluminum samples increases throughout the studied range of modifier concentrations. With a 50% modifier content, the shear strength increases by 127% (EDEGA), 154% (EDEGS), and 188% (EDEGT) compared to the initial polymer.

Thus, good compatibility between the reactive modifier and the epoxy matrix has a significant positive effect on the mechanical properties of the modified polymers.

## 4. Conclusions

It is shown that oligoesters based on adipic, sebacic, and tetradecanedioic acids with reactive carboxyl groups, as well as their epoxy resin adducts with reactive epoxy groups, were synthesized without the use of catalysts and solvents. The preparation of epoxy polymers modified by oligoesters with epoxy reactive groups was also environmentally safe.

The single-phase structure of the modified polymers was confirmed using the DMA, TGA, and SEM methods. It is shown that for the modified polymers, a pattern of plastic deformation is observed, in contrast to the brittle destruction of the initial polymer.

The observed effect of a significant increase in the dynamic modulus of elasticity of modified polymers below 25 °C can be explained by the fact that flexible modifier molecules are able to form a more densely packed polymer in their glassy state.

The mechanical properties of the polymers based on mixtures of epoxy resin CYD-128 and the synthesized modifiers have been systematically studied in a wide range of concentrations. The modifying effect is observed when using all the synthesized oligoesters with epoxy reactive groups in all the studied concentration ranges. At the same time, it is possible to obtain epoxy polymers with the required properties by regulating the amount of the introduced modifier.

It has been found that elongation at break, impact strength, work of fracture, and shear strength increase monotonously throughout the studied concentration range (at 50% modifier content, elongation at break and shear strength increase by ~450% and ~150%, respectively, compared with an unmodified polymer.

The results obtained demonstrate that synthesized modifiers with reactive epoxy groups can contribute to the creation of new cold-cured epoxy materials with an improved complex of properties for various industries.

## Figures and Tables

**Table 1:** Glass transition temperature of epoxy polymers containing 20% and 50% of modifier.

**Modifier Content, %**	0%	20% EDEGA	20% EDEGS	20% EDEGT	50% EDEGA	50% EDEGS	50% EDEGT
**T_g_, °C**	89.64	83.11	87.88	88.94	50.19	60.33	69.16

**Table 3:** Temperature of 5% mass loss of epoxy polymers containing 20% and 50% of modifier.

**Modifier Content, %**	0%	20% EDEGA	20% EDEGS	20% EDEGT	50% EDEGA	50% EDEGS	50% EDEGT
**T_5%_, °C**	406.31	375.84	390.04	384.71	365.57	361.97	370.59

**Table 4:** The effect of the concentration of modifiers on the bending strength.

Modifier Content, %	Bending Strength, MPa		
	EDEGA	EDEGS	EDEGT
0	80.49	80.49	80.49
1.5	86.65	87.27	88.82
5	95.29	97.67	102.92
10	77.78	86.26	Not Broken
20	60.61	72.63	Not Broken
50	Not Broken	Not Broken	Not Broken

**Table 2:** Storage modulus of epoxy polymers containing 20% and 50% of modifier.

Modifier Content, %	Storage Modulus, MPa	
	−100 °C	20 °C
0%	1903.78	1357.15
20% EDEGA	2261.95	1504.61
20% EDEGS	3378.35	2179.23
20% EDEGT	3869.69	2314.25
50% EDEGA	3376.17	719.93
50% EDEGS	3675.76	1017.52
50% EDEGT	3704.74	1510.65

## Data Availability

The authors confirm that the data supporting the findings of this study are available within the article.
